# Reviewers in 2021

**DOI:** 10.1098/rsbl.2022.0069

**Published:** 2022-03-16

**Authors:** David J. Beerling

**Affiliations:** *Editor-in-Chief*

Despite the continued difficulties that have come with the pandemic, our peer reviewers once again stepped up to support *Biology Letters*. The vital feedback that reviewers provided authors enabled us to continue publishing high-quality articles that benefit the entire scientific community. The Editors of the Journal would therefore like to express their sincere thanks to all those who contributed their time by refereeing manuscripts submitted throughout 2021.

We know that most reviewers value recognition of their efforts by the journal and through services, such as Publons, which is integrated with *Biology Letters*. To provide an opportunity for recognition, we also list the names of all reviewers (unless they have opted out). This article is made permanent and citable by its digital object identifier (DOI). We encourage you to quote your contribution in your applications for tenure and grants or other forms of research assessment. Where you have provided it, we have included your ORCID, so your contribution can be unambiguously assigned to you.

As always, the team really does appreciate all the work our reviewers do on behalf of the journal, and we look forward to working with you again in 2022.

Abacı SH
Abdai J https://orcid.org/0000-0003-1219-2858
Abdala V
Abe M https://orcid.org/0000-0002-0468-1179
Abram P
Ache J
Adam T
Adams D
Adams R
Aisenberg A https://orcid.org/0000-0002-1139-6480
Akcali C https://orcid.org/0000-0002-8089-3501
Alcover J
Allu P
Alton L https://orcid.org/0000-0002-4236-2494
Alvarez-Sanchez ME https://orcid.org/0000-0002-9212-4451
Amaya-Márquez M https://orcid.org/0000-0001-6135-8063
Amo L https://orcid.org/0000-0003-4621-3493
Amoroso C https://orcid.org/0000-0001-5456-7975
Andrews C
Andrews K
Aonuma H https://orcid.org/0000-0001-8380-1820
Arcese P
Arranz I
Arvanitidis C
Assis V
Aubier T https://orcid.org/0000-0001-8543-5596
Auger-Méthé M https://orcid.org/0000-0003-3550-4930
Aureli F https://orcid.org/0000-0002-0671-013X
Austin J https://orcid.org/0000-0003-4244-2942
Avila Segura L https://orcid.org/0000-0003-1642-3537
Avila I
Axmacher J
Baack E
Baddeley R
Baird A
Baird R
Baj A https://orcid.org/0000-0003-0088-2712
Bajer A
Balenger S
Ball M
Balshine S
Baniaga A
Barata C
Barbaro L https://orcid.org/0000-0001-7454-5765
Barclay K https://orcid.org/0000-0001-8075-6948
Barraquand F https://orcid.org/0000-0002-4759-0269
Barrett B https://orcid.org/0000-0002-2725-2385
Bartley M
Bartlow A
Barton R
Bartonička T
Bastos R
Bauch C https://orcid.org/0000-0003-0218-5582
Bauman A https://orcid.org/0000-0001-9260-2153
Bear M
Beardsworth C https://orcid.org/0000-0003-1308-1455
Beatty C
Beauchamp G https://orcid.org/0000-0002-3393-9562
Becker DJ
Becklin K
Belasen A https://orcid.org/0000-0002-1306-3436
Béliveau A
Belliure J
Belmaker J https://orcid.org/0000-0002-5618-7359
Belmonte G
Benson T
Benton M https://orcid.org/0000-0002-4323-1824
Bentsen S
Beran M https://orcid.org/0000-0002-2504-0338
Berenbaum M https://orcid.org/0000-0003-3165-8385
Bernardo J
Berteaux D
Bertelsmeier C https://orcid.org/0000-0003-3624-1300
Berthet M
Bibby K
Biewener A https://orcid.org/0000-0003-3303-8737
Blanke A
Bo W https://orcid.org/0000-0001-5303-4114
Boersch-Supan P https://orcid.org/0000-0001-6723-6833
Bogdanowicz W
Bonduriansky R https://orcid.org/0000-0002-5786-6951
Bongers F
Bonneaud C https://orcid.org/0000-0003-2248-3288
Booth W https://orcid.org/0000-0003-2355-0702
Boratynski J https://orcid.org/0000-0002-2442-7709
Borremans B https://orcid.org/0000-0002-7779-4107
Bortolotto G https://orcid.org/0000-0002-5343-6575
Bortolus A
Boss E
Bourgeois Y https://orcid.org/0000-0002-1809-387X
Bowman A
Bradfield J
Branch C https://orcid.org/0000-0003-1769-5040
Brancini G
Brandl H
Brandon C
Bray G
Breed W
Breen A https://orcid.org/0000-0002-2331-0920
Brewster L
Bridge T https://orcid.org/0000-0003-3951-284X
Briedis M
Brigham R
Briskie J
Britz R https://orcid.org/0000-0002-0126-4660
Brodin A https://orcid.org/0000-0001-5798-888X
Broggi J https://orcid.org/0000-0002-1706-4014
Brougham T
Brown G
Brown M
Brückner A https://orcid.org/0000-0002-9184-8562
Brunton D
Buczkowski G
Burdfield-Steel E
Burgess E
Burghardt G
Burns K https://orcid.org/0000-0002-4938-2877
Burrows A
Bush S
Butterworth B https://orcid.org/0000-0001-8201-3347
Byrne M https://orcid.org/0000-0002-8902-9808
Byrnes E
C. Lopes P https://orcid.org/0000-0001-5170-3230
Cabirol A
Calovi D https://orcid.org/0000-0002-2452-9801
Camacho A https://orcid.org/0000-0003-2978-792X
Camacho-Sanchez M https://orcid.org/0000-0002-6385-7963
Campany C
Campiao K
Campos R
Camus M https://orcid.org/0000-0003-0626-6865
Cannatella D https://orcid.org/0000-0001-8675-0520
Card D
Cardinale M
Cardoso D https://orcid.org/0000-0002-2811-2536
Cardoso G https://orcid.org/0000-0001-6258-1881
Careau V https://orcid.org/0000-0002-2826-7837
Carlson B
Carnevale G
Caro S https://orcid.org/0000-0002-5405-7753
Caro S
Carretero M
Carrier D
Carter G https://orcid.org/0000-0001-6933-5501
Caruana F https://orcid.org/0000-0002-4631-7543
Carvajal-Rodríguez A
Casas J https://orcid.org/0000-0003-1666-295X
Castonguay M
Cau A
Caves E https://orcid.org/0000-0003-3497-5925
Cerri M https://orcid.org/0000-0003-3556-305X
Chacron M
Chang C-W https://orcid.org/0000-0002-9817-2956
Chang H
Chapman S https://orcid.org/0000-0002-5825-2329
Chapman T
Chattopadhyay J
Chen G
Chimienti M
Chisholm R
Chomicki G
Choo B
Christophe P https://orcid.org/0000-0001-6425-9060
Cieri R https://orcid.org/0000-0001-6905-9148
Clark C https://orcid.org/0000-0002-6311-9768
Clark T
Clay T
Clayton N https://orcid.org/0000-0003-1835-423X
Clegg S
Clutton-Brock T https://orcid.org/0000-0001-8110-8969
Coates M https://orcid.org/0000-0003-2843-1075
Cobley P
Colston T https://orcid.org/0000-0002-1127-1207
Connallon T https://orcid.org/0000-0002-1962-4951
Connolly S
Cooper I
Cornell A https://orcid.org/0000-0002-8782-084X
Correa-Garhwal S
Costilla R https://orcid.org/0000-0003-0818-5065
Cox C https://orcid.org/0000-0002-9424-8482
Crates R
Crespi B https://orcid.org/0000-0002-0362-392X
Cristiano M
Crompton A
Cronin A https://orcid.org/0000-0003-2075-1138
Cross E https://orcid.org/0000-0002-1671-5698
Crossley D
Cryan P
Culina A https://orcid.org/0000-0003-2910-8085
Cullen T https://orcid.org/0000-0002-4261-1323
Currie C
Cuthbert R https://orcid.org/0000-0003-2770-254X
Cuthill I https://orcid.org/0000-0002-5007-8856
Cuturi L
da Rocha JBT
Dajoz I
D'Alba L https://orcid.org/0000-0002-2478-3455
Dallinger R
Darragh K
Darroch S
Das M
Daversa D https://orcid.org/0000-0002-8984-8897
Davidson G
Davis Rabosky A
De Lisle S https://orcid.org/0000-0001-9587-8665
de Lourdes Serrano Sánchez M
de Margerie E https://orcid.org/0000-0002-5380-3355
de Oliveira E
De Paula Oliveira T
de Vos J
Debarre F https://orcid.org/0000-0003-2497-833X
DeBiasse M
Deboer T
DeCasien A https://orcid.org/0000-0002-6205-5408
Deeming DC https://orcid.org/0000-0002-9587-6149
Dehning J https://orcid.org/0000-0002-1728-2505
Delabie J
Desjonquères C https://orcid.org/0000-0002-6150-3264
Diego-Rasilla FJ https://orcid.org/0000-0002-7682-014X
Dillon M
Dingemanse N
Doherty J-F https://orcid.org/0000-0003-4766-9417
Domenici P https://orcid.org/0000-0003-3182-2579
Dominy N https://orcid.org/0000-0001-5916-418X
Dougherty L https://orcid.org/0000-0003-1406-0680
Downie JR
Drake A
Drobniak S
Duffy M https://orcid.org/0000-0002-8142-0802
Dunlap A https://orcid.org/0000-0003-2429-5758
Dunn P
Dupoué A https://orcid.org/0000-0002-2501-464X
Dupuis J
Durantini E
Durbach I
Duso C
Eberhard W
Eccles M
Edinborough K
Eisenberg D https://orcid.org/0000-0003-0812-1862
Eldridge D
Elfenbein HA https://orcid.org/0000-0002-3973-1447
Elosegui A
Endler J https://orcid.org/0000-0002-7557-7627
Engelberth J
Ermolaeva M
Ernst J
Fabbri M https://orcid.org/0000-0002-1257-1594
Fabre A-C https://orcid.org/0000-0001-7310-1775
Fabry B
Fahad S
Fahrbach S
Fang S
Farber S
Faria G
Farina A
Farina SC https://orcid.org/0000-0003-2479-1268
Farine D https://orcid.org/0000-0003-2208-7613
Felsenberg J
Ferdy J-B
Ferreira R https://orcid.org/0000-0001-7774-5252
Ferreira T
Ferrón H https://orcid.org/0000-0001-5183-7220
Festa-Bianchet M
Fiedler K
Fiksen Ø
Fischer S
Fisher H https://orcid.org/0000-0001-5622-4335
Fjeldså J
Fleishman L
Florez L
Forbes M
Forstmeier W https://orcid.org/0000-0002-5984-8925
Fraley G
Franks N https://orcid.org/0000-0001-8139-9604
Fraser D https://orcid.org/0000-0002-0228-2617
Frasier T https://orcid.org/0000-0002-3055-0199
Freeman N https://orcid.org/0000-0002-8871-2963
Freitas M
Frost C
Frynta D https://orcid.org/0000-0002-1375-7972
Fujita M
Gagliardo A https://orcid.org/0000-0003-0214-3817
Gaillard J-M
Gaitan-Espitia J https://orcid.org/0000-0001-8781-5736
Galdino C https://orcid.org/0000-0001-8209-3121
Galipaud M
Gamperl K
Gangloff E https://orcid.org/0000-0002-9074-8009
García-Roger E
Garner T https://orcid.org/0000-0002-0336-9706
Garnier S https://orcid.org/0000-0002-3886-3974
Gasparini J
Gaston K
Gehman A-L https://orcid.org/0000-0003-0595-9950
Germain D https://orcid.org/0000-0002-7608-2212
Getz W https://orcid.org/0000-0001-8784-9354
Ghasemi S
Gifford R https://orcid.org/0000-0003-4028-9884
Gilmour A
Giraudeau M https://orcid.org/0000-0001-8563-1810
Girondot M https://orcid.org/0000-0001-6645-8530
Gleichsner A
Glendinning J
Glimm T
Goblirsch M
Gomez-Marin A https://orcid.org/0000-0003-2764-2583
Gomez-Samblas M
González-Irusta JM https://orcid.org/0000-0002-3948-604X
Goodman S
Gotoh T
Gould S
Gray D https://orcid.org/0000-0002-4439-561X
Green J
Greenwold M
Greig D
Grieves L https://orcid.org/0000-0002-6836-2177
Grollemund P-M https://orcid.org/0000-0002-1273-1658
Gronenberg W
Gruber T https://orcid.org/0000-0002-6766-3947
Grunst M https://orcid.org/0000-0002-3425-4020
Grüter C
Gubert P
Guglielmo C
Guill C
Guillette L https://orcid.org/0000-0002-8777-6543
Guleria P https://orcid.org/0000-0001-5235-4829
Gundiah N https://orcid.org/0000-0002-9882-5232
Guo B
Guo C
Gurney J
Gutierrez J-C
Gutiérrez-Cánovas C
Halley J
Halliday W
Hammer Ø
Hammerschmidt K
Hand S
Hanke F
Hanlon R https://orcid.org/0000-0003-0004-5674
Hardy I https://orcid.org/0000-0002-5846-3150
Haridy Y https://orcid.org/0000-0002-6908-5749
Haro A
Haruda A
Hassenklöver T
Hastie G https://orcid.org/0000-0002-9773-2755
Hastings J
Hauber M https://orcid.org/0000-0003-2014-4928
Havlíček J https://orcid.org/0000-0003-2374-7337
Hawkins A
Hayashi M
Hayden B https://orcid.org/0000-0002-7678-4281
Hayes J
Healy SD
Hedenström A https://orcid.org/0000-0002-1757-0945
Hedouin L https://orcid.org/0000-0001-6565-3633
Hegemann A https://orcid.org/0000-0002-3309-9866
Heidinger B https://orcid.org/0000-0003-0064-209X
Hejnol A https://orcid.org/0000-0003-2196-8507
Helle S https://orcid.org/0000-0002-6216-3759
Hellgren O
Helm B https://orcid.org/0000-0002-6648-1463
Helms J
Hempel de Ibarra N https://orcid.org/0000-0002-0859-8217
Hennin H
Hermann S
Hernández-León S
Hickey A https://orcid.org/0000-0003-3784-4816
Higgs D https://orcid.org/0000-0002-0771-4642
Higham J https://orcid.org/0000-0002-1133-2030
Hill R https://orcid.org/0000-0002-7601-5802
Hill S
Hinde K
Hines H https://orcid.org/0000-0003-0299-5569
Ho S
Hochkirch A
Hodkinson I
Hoffmann S
Holden W
Holt R https://orcid.org/0000-0002-6685-547X
Hone D
Hongoh Y
Hopper L https://orcid.org/0000-0001-5953-3264
Hopwood C
Horne D
Horschler D https://orcid.org/0000-0003-0440-8217
Hortal J
Houde P
Howard C https://orcid.org/0000-0001-7514-9721
Huang Q https://orcid.org/0000-0001-5528-5099
Hubert P
Huey R https://orcid.org/0000-0002-4962-8670
Huffard C
Huppop O
Husak J https://orcid.org/0000-0002-5860-3774
Hussey N https://orcid.org/0000-0002-9050-6077
Iguchi A https://orcid.org/0000-0002-8894-1977
Immler S https://orcid.org/0000-0003-1234-935X
Ince L
Ingle D
Innocenti F
Ioannou C https://orcid.org/0000-0002-9739-889X
Irving T
Isaac N
Isaza R
Isojunno S https://orcid.org/0000-0002-2212-2135
Izzo T
Jaeger J-J
James P
Janko K
Jarčuška B https://orcid.org/0000-0002-0654-9171
Jarvi S
Jayne B
Jeglinski J https://orcid.org/0000-0002-7997-6756
Jennings D https://orcid.org/0000-0001-5369-3574
Jewell J
Jiang P
Jiao H
Johnson B
Johnson J
Johnston T
Jokela J
Jones C
Jones A
Jongejans E
Jonkers L https://orcid.org/0000-0002-0253-2639
Jonsson C
Joyce P https://orcid.org/0000-0003-1058-7901
Juen L https://orcid.org/0000-0002-6188-4386
Kalinkat G https://orcid.org/0000-0003-3529-5681
Kalyanasundaram P
Karanth P
Kardynal K
Katzir G
Kaunisto K https://orcid.org/0000-0001-6665-0047
Kelley J https://orcid.org/0000-0002-8223-7241
Kelly D
Kenchington E
Kennett D
Khadempour L https://orcid.org/0000-0002-7043-3720
Kier W
Kiflawi M https://orcid.org/0000-0002-9705-6486
Killen S https://orcid.org/0000-0003-4949-3988
Kirk D https://orcid.org/0000-0001-9588-1004
Kirkpatrick L https://orcid.org/0000-0002-0161-2469
Kishkinev D https://orcid.org/0000-0002-2619-1197
Kissane R https://orcid.org/0000-0001-9385-2584
Klaczko L
Klein N
Klofstad C
Klokočovnik V
Klümper U https://orcid.org/0000-0002-4169-6548
Kobayasi K
Kohlmeier P
Koppik M
Korpimäki E https://orcid.org/0000-0001-7596-1955
Krasheninnikova A https://orcid.org/0000-0001-8566-8277
Krause T https://orcid.org/0000-0002-8327-3711
Kremen C
Kremp A https://orcid.org/0000-0001-9484-6899
Kretschmer R
Krupandan E https://orcid.org/0000-0002-0654-9745
Ksepka D
Kukekova A
Kültz D
Kurganov E
Kusch J https://orcid.org/0000-0003-0078-5621
Labra A
Lach L https://orcid.org/0000-0001-5137-5185
Lailvaux S
Lam F-P
LaManna J
Lambrechts M
Lanctot R
Lankheet M https://orcid.org/0000-0002-8261-2187
Lanyon J
LaPointe D
Larsen O https://orcid.org/0000-0002-8325-0982
Larson E
Laskowski K https://orcid.org/0000-0003-1523-9340
Lawrence AB
Le Croizier G https://orcid.org/0000-0002-5093-8406
Leather S
Lee C-Y
Lee J-S
Leighton G https://orcid.org/0000-0003-0564-3980
Lemaire BS
Letessier T https://orcid.org/0000-0003-4011-0207
Levesque D https://orcid.org/0000-0003-0132-8094
Lewis C https://orcid.org/0000-0002-3564-2906
Liebhold A
Liew T-S
Light T
Ligocki I
Lim M
Lima C https://orcid.org/0000-0003-3058-7204
Linck E
Lindemann J
Link A
Liszka C https://orcid.org/0000-0003-1309-4045
Little T
Liu C
Liu Y https://orcid.org/0000-0003-3948-1246
Llaurens V
Lo N https://orcid.org/0000-0003-2176-2840
Logue D
Lohan K
Lomolino M
Longden K
López Garrido J
Lopez Sepulcre A
Lorch J
Loughman Z
Loukola O https://orcid.org/0000-0002-9094-2004
Lu J https://orcid.org/0000-0002-5791-4749
Ludsin S https://orcid.org/0000-0002-3866-2216
Luijckx P
Lüpold S https://orcid.org/0000-0002-5069-1992
Ma C
Maas H
Macgregor C https://orcid.org/0000-0001-8281-8284
Machanda Z https://orcid.org/0000-0001-7060-7949
Machlus K
Machovsky-Capuska G
Macias-Macias JO
MacKay D
MacNulty D
Madau F
Maggini I https://orcid.org/0000-0002-1528-2288
Mainwaring M https://orcid.org/0000-0002-0427-9673
Maisch T
Mann R https://orcid.org/0000-0003-0701-1274
Manthey J
Markow T
Marley S https://orcid.org/0000-0001-5950-4949
Marsland S https://orcid.org/0000-0002-9568-848X
Martin G
Martin R https://orcid.org/0000-0002-1155-6704
Martins MJF
Masek P
Masiero S
Massa B
Matich P
Matsuzawa T https://orcid.org/0000-0002-8147-2725
Matthews C https://orcid.org/0000-0002-8608-3905
Matyas G
Mayhew J
Mazzoni V https://orcid.org/0000-0003-4036-5322
McCabe B
McCauley S https://orcid.org/0000-0001-9649-6693
McClure M https://orcid.org/0000-0003-3590-4002
McComas K
McConkey K
McCoy D https://orcid.org/0000-0001-8383-8084
McGaw I https://orcid.org/0000-0001-7854-0216
McKechnie A https://orcid.org/0000-0002-1524-1021
McLeod A
McMahon B https://orcid.org/0000-0003-3143-8075
Meegaskumbura M https://orcid.org/0000-0002-4599-6724
Meier R https://orcid.org/0000-0002-4452-2885
Memery L
Menzel F https://orcid.org/0000-0002-9673-3668
Meredith R
Merilä J https://orcid.org/0000-0001-9614-0072
Merilaita S https://orcid.org/0000-0001-6437-9863
Merullo D
Mesoudi A https://orcid.org/0000-0002-7740-1625
Meuthen D https://orcid.org/0000-0002-3373-5383
Michalczyk Ł https://orcid.org/0000-0002-2912-4870
Michalska K https://orcid.org/0000-0002-5136-7836
Mikheyev A
Minias P https://orcid.org/0000-0002-7742-6750
Mitchell K https://orcid.org/0000-0002-3921-0262
Mitchell N https://orcid.org/0000-0003-0744-984X
Miyatake T https://orcid.org/0000-0002-5476-0676
Moeller A
Mohammadi S https://orcid.org/0000-0003-3450-6424
Moleón M https://orcid.org/0000-0002-3126-619X
Molnar C
Moore M https://orcid.org/0000-0002-8255-4247
Morand S https://orcid.org/0000-0003-3986-7659
Morand-Ferron J https://orcid.org/0000-0001-5186-7710
Morawo T
Moreau C https://orcid.org/0000-0003-1139-5792
Moreno-Letelier A https://orcid.org/0000-0001-7524-7639
Moreno-Rueda G https://orcid.org/0000-0002-6726-7215
Morris J https://orcid.org/0000-0002-8647-4420
Morrissey C
Mortier F https://orcid.org/0000-0002-1480-2675
Moskat C https://orcid.org/0000-0002-8800-4975
Motani R https://orcid.org/0000-0001-5022-1053
Mourier J https://orcid.org/0000-0001-9019-1717
Müller R https://orcid.org/0000-0001-8894-9875
Muralidhar P
Murphy P
Musiani M
Nager R
Namgail T
Nauen R
Navalón G https://orcid.org/0000-0002-2447-1275
Navarro N
Navedo JG https://orcid.org/0000-0003-3451-1792
Nedoluzhko A https://orcid.org/0000-0001-7040-0892
Neilly H
Nesher N
Nespolo R https://orcid.org/0000-0001-9337-0606
Neufer PD
Nevo O https://orcid.org/0000-0003-3549-4509
Newton I
Ngamniyom A
Nganvongpanit K
Nicholls E https://orcid.org/0000-0002-3697-0223
Nicieza A https://orcid.org/0000-0003-4062-569X
Nieder A https://orcid.org/0000-0001-6381-0375
Nilsson RH
Noakes M
Noguerales V
Nol E
Nonacs P https://orcid.org/0000-0003-3010-0777
Norfolk O
Novak C
Nowicki S https://orcid.org/0000-0002-6564-905X
O'Connell L https://orcid.org/0000-0002-2706-4077
Odetokun I https://orcid.org/0000-0002-5171-1895
O'Donnell S https://orcid.org/0000-0002-6338-7148
Oduor A https://orcid.org/0000-0001-6714-5253
O'Hara M
Olli K
Olofsson J https://orcid.org/0000-0002-6943-1218
Olson ME
Olsson M https://orcid.org/0000-0002-4130-1323
Ortiz-Ceballos A https://orcid.org/0000-0001-8700-4503
Orton R https://orcid.org/0000-0002-3389-4325
Osiejuk T
Osland M
Ostojić L
Page L
Palacios O
Palaoro A https://orcid.org/0000-0002-8629-0728
Palavalli-Nettimi R
Palme R
Park K
Parker P
Parrott B
Pasquesi G https://orcid.org/0000-0002-3400-8057
Pavan G
Paxton C https://orcid.org/0000-0002-9350-3197
Pearce-Higgins J
Pedersen A
Pedersen E
Peel A
Pelster B
Pennell T
Peona V https://orcid.org/0000-0001-5119-1837
Pepperberg I https://orcid.org/0000-0001-9486-7323
Pernet B
Peron G https://orcid.org/0000-0002-6311-4377
Petillon J
Photopoulou T https://orcid.org/0000-0001-9616-9940
Pietri J
Pilaar Birch S
Pincebourde S
Pintar M https://orcid.org/0000-0003-0165-3882
Piorkowski D https://orcid.org/0000-0003-1405-1020
Pirk C https://orcid.org/0000-0001-6821-7044
Piscopo M
Pitts-Singer T
Podos J
Poggiale J-C
Polese G
Potapov A
Potier S
Powell S
Power E https://orcid.org/0000-0002-3064-2050
Pradhan N
Pratt RB
Pravosudov V https://orcid.org/0000-0003-1117-7875
Prendini L
Presley S
Pringle E https://orcid.org/0000-0002-4398-9272
Prior K
Prokkola J https://orcid.org/0000-0003-2987-4417
Prum R
Pruvot M
Pull C
Purse B
Putman N https://orcid.org/0000-0001-8485-7455
Quillfeldt P
Råberg L https://orcid.org/0000-0001-5219-7448
Raguso R https://orcid.org/0000-0003-2066-3269
Raine N https://orcid.org/0000-0001-6343-2829
Ramenofsky M
Ramm S https://orcid.org/0000-0001-7786-7364
Randall C
Raybould A
Regular P
Restif O https://orcid.org/0000-0001-9158-853X
Reuter M
Reznikova Z
Riesch R https://orcid.org/0000-0002-0223-1254
Ringler E https://orcid.org/0000-0003-3273-6568
Ritchie M
Rittschof C https://orcid.org/0000-0003-0808-0458
Riva F https://orcid.org/0000-0002-1724-4293
Roberts M https://orcid.org/0000-0003-2693-5093
Roberts W
Robertson G https://orcid.org/0000-0001-8452-5506
Robertson J
Robinson K https://orcid.org/0000-0002-6212-9710
Rode N https://orcid.org/0000-0002-1121-4202
Roeder K https://orcid.org/0000-0002-2628-5003
Rohani P https://orcid.org/0000-0002-7221-3801
Rojas B https://orcid.org/0000-0002-6715-7294
Rojas D https://orcid.org/0000-0002-5835-0324
Roll U
Romero T
Romiguier J
Rosenthal G
Ross J https://orcid.org/0000-0001-7413-4704
Rossi S
Rossie J
Rovinsky D https://orcid.org/0000-0003-4356-9523
Rowcliffe M
Rubolini D
Rueger T https://orcid.org/0000-0002-2105-6412
Ruggiero R
Russo T
Saccheri I https://orcid.org/0000-0003-0476-2347
Saito K
Saitoh K
Sakamoto T
Sakiyama T https://orcid.org/0000-0002-2687-7228
Salvador R https://orcid.org/0000-0002-4238-2276
Samplonius JM
Sander PM
Sandkam B https://orcid.org/0000-0002-5043-9295
Sasaki T https://orcid.org/0000-0001-7923-9855
Sawtell N
Schaefer R
Schafer T
Schausberger P https://orcid.org/0000-0002-1529-3198
Scheiner S
Schlenke T
Schmitz O https://orcid.org/0000-0003-1515-2667
Schoeman D
Schoepf I https://orcid.org/0000-0001-5437-337X
Schramm Y
Schülke O https://orcid.org/0000-0003-0028-9425
Schulte L
Schulte P https://orcid.org/0000-0002-5237-8836
Schultheiss P https://orcid.org/0000-0002-6906-3507
Schulz-Kornas E https://orcid.org/0000-0003-1657-8256
Schwarzenberger A
Schweder T
Schweinfurth M https://orcid.org/0000-0003-2066-7892
Sedio BE
Segura D
Seid M
Selden P
Serrao JE https://orcid.org/0000-0002-0477-4252
Shafir S
Shapiro O
Sherratt E https://orcid.org/0000-0003-2164-7877
Sherry D
Sherwin W
Sheth S
Shine R https://orcid.org/0000-0001-7529-5657
Shiojiri K
Shu-Chien AC
Shuker D
Shuter B
Siciliano S
Siegel D
Sigwart J https://orcid.org/0000-0002-3005-6246
Sih A
Silby M
Silva D
Silveira L
Simmons N
Simons M
Singh B
Skiba S https://orcid.org/0000-0002-0014-099X
Slotnick B
Ślusarczyk M https://orcid.org/0000-0002-2311-5033
Smit B
Smith AR https://orcid.org/0000-0002-4291-6928
Smith J https://orcid.org/0000-0002-3342-4454
Smulders T https://orcid.org/0000-0002-6673-7596
Sneddon L
Soares L https://orcid.org/0000-0002-6933-8048
Sockman K https://orcid.org/0000-0002-3846-5386
Sol D https://orcid.org/0000-0001-6346-6949
Soler J https://orcid.org/0000-0003-2990-1489
Solow A https://orcid.org/0000-0003-1978-1372
Somanathan H https://orcid.org/0000-0002-4803-3894
Somjee U https://orcid.org/0000-0002-5130-3605
Song C https://orcid.org/0000-0001-7490-8626
Sprayberry JDH
Staples T
Stavenga D https://orcid.org/0000-0002-2518-6177
Steidle J https://orcid.org/0000-0003-2386-3063
Stephan J https://orcid.org/0000-0001-6195-7867
Stephenson J https://orcid.org/0000-0001-8939-5149
Stevenson P
Stockl A
Stork N
Straley J
Strand M https://orcid.org/0000-0003-1844-7460
Strauss J
Sturdy C
Sudyka J
Šulc M https://orcid.org/0000-0002-7866-1795
Sundstrom F https://orcid.org/0000-0002-3157-7289
Szydlo W
Takami Y https://orcid.org/0000-0002-6507-2115
Tarvin R
Tatarnic N https://orcid.org/0000-0001-8641-4412
Taylor A
Taylor R https://orcid.org/0000-0003-3173-5432
Taylor-Rodriguez D
Tecwyn E
Tekle Y https://orcid.org/0000-0003-2781-2351
Tekwa E https://orcid.org/0000-0003-2971-6128
Tell L
Tellier A https://orcid.org/0000-0002-8895-0785
Tello-Ramos M https://orcid.org/0000-0003-0945-3498
Tew N
Theobald J
Thibodeau P
Thiel M
Thiery G https://orcid.org/0000-0003-4789-7372
Thogmartin W https://orcid.org/0000-0002-2384-4279
Thompson C
Thomsen M
Thomson J
Thomson V https://orcid.org/0000-0001-8368-9664
Thorogood R https://orcid.org/0000-0001-5010-2177
Tian J
Tickle P
Tomlinson S https://orcid.org/0000-0003-0864-5391
Tonnabel J
Travis J https://orcid.org/0000-0002-5785-4272
Tregenza T https://orcid.org/0000-0003-4182-2222
Treves A https://orcid.org/0000-0002-3052-4708
Triki Z https://orcid.org/0000-0001-5592-8963
Trinajstic K
Trivedi A https://orcid.org/0000-0003-0985-3088
Trumbore S
Tschirren B https://orcid.org/0000-0003-4806-4102
Tsukaya H
Tunnicliffe V
Turner E
Twiss S https://orcid.org/0000-0002-1923-8874
Upham N
Urban M https://orcid.org/0000-0003-3962-4091
Üveges B
Vale G https://orcid.org/0000-0001-5228-439X
Van Damme R
van Heerwaarden B
van Klink R https://orcid.org/0000-0002-8125-1463
Van Opzeeland I
van Velzen E
Van Wassenbergh S https://orcid.org/0000-0001-5746-4621
Varnon C
Vasemägi A
Vatandoost H https://orcid.org/0000-0002-5983-9420
Vedder O
Videvall E https://orcid.org/0000-0002-9998-3689
Vieira-Potter V
Vitule JR
Vizentin-Bugoni J https://orcid.org/0000-0002-6343-3650
Vogels R
Vogler A
Walker R
Wang Y https://orcid.org/0000-0003-3743-3937
Warne M
Warrant E https://orcid.org/0000-0001-7480-7016
Warren R
Waters J https://orcid.org/0000-0002-1514-7916
Watson S
Watts H https://orcid.org/0000-0002-9888-825X
Watts P
Webb C
Weber M
Wegner N
Weir L
Weithoff G
Werner C https://orcid.org/0000-0002-0442-9811
Wheatcroft D
White O
Whitney N
Willett C
Williams R
Williams S
Willink B https://orcid.org/0000-0002-4579-6909
Wilson Rankin E
Wilson R https://orcid.org/0000-0003-3177-0107
Winters A https://orcid.org/0000-0003-2840-6267
Wolinska J
Wong M https://orcid.org/0000-0001-6393-6453
Wood J
Woods HA
Worthy TH https://orcid.org/0000-0001-7047-4680
Wright SJ
Wykowska A https://orcid.org/0000-0003-3323-7357
Yap KN
Yoccoz N
Yoda K
Yoon K
Young K
Yuda P
Yusuf A https://orcid.org/0000-0002-8625-6490
Zajitschek S https://orcid.org/0000-0003-4676-9950
Zawierucha K
Zelditch M https://orcid.org/0000-0002-5006-6679
Zeman M
Zeppelini C https://orcid.org/0000-0002-0490-4395
Zhang Y-Y https://orcid.org/0000-0002-8636-2242
Zhong Z
Zmudzki J
Züst T https://orcid.org/0000-0001-7142-8731

